# Supplementary material for: Chemical control of excited-state reactivity of the anionic green fluorescent protein chromophore
Source: Commun Chem. 2024 Feb 5;7:25. doi: 10.1038/s42004-024-01099-1 (PMC10844232; doi:10.1038/s42004-024-01099-1)
Supplement: Supplementary file 2 — Supplementary material [file 42004_2024_1099_MOESM2_ESM.pdf]

Supplementary Information:
Chemical Control of Excited-State Reactivity of the Anionic Green Fluorescent Protein Chromophore

Nanna H. List,[1#] Chey M. Jones[2,3] and Todd J. Martínez[2,3*]
[1]Department of Chemistry, KTH Royal Institute of Technology, SE-10044 Stockholm, Sweden
[2]Department of Chemistry and The PULSE Institute, Stanford University, Stanford, CA 94305
[3]SLAC National Accelerator Laboratory, 2575 Sand Hill Road, Menlo Park, CA 94025

[#]nalist@kth.se
[*]toddjmartinez@gmail.com

Contents (Supplementary X)

Three-state diabatic model	Methods 1
Cone sampling and photoisomerization committor	Methods 2
Validation of α-CASSCF against XMS-CASPT2	Note 1
Orientation of methoxy group in MHBDI⁻	Note 2
Atom labeling and definition of key geometric parameters	Figure 1
Active-space orbitals and corresponding Boys-localized orbitals	Figure 2
Three-state diabatic model	Figure 3
Comparison of α-CASSCF and XMS-CASPT2: relative energies at key geometries	Figure 4
Substituent effects at the FC point within the three-state diabatic model	Figure 5
Relaxed scan of methoxy orientation in MHBDI⁻	Figure 6
Progress of the S_1 wavepacket along the I-torsional and HOOP modes	Figure 7
I- and P-torsional distributions of the spawning geometries	Figure 8
Topography and electronic character of MECIs	Figure 9
Comparison of α-CASSCF and XMS-CASPT2: relative energies at critical points	Tables 1-4
Substituent effects at the FC point	Table 5
Selected geometric parameters for critical points at the α-CASSCF level	Tables 6-9
Selected geometric parameters for critical points at the XMS-CASPT2 level	Tables 10-13
Mulliken charges for key critical points	Table 14
Intersection parameters for MECIs	Table 15
Effects of geometric deformations on the elements of the three-state diabatic Hamiltonian	Tables 16-17

References

Supplementary Methods 1: Three-state diabatic model

To guide the choice of chemical modification, we use the three-state diabatic model proposed by Olsen and McKenzie.[1] This model represents an adiabatic-to-quasi-diabatic transformation that combines Boys orbital localization of the converged activate-space orbitals and unitary block-diagonalization[2,3] to yield charge-localized quasi-diabatic states. Specifically, as shown in the original work by Olsen and McKenzie, Boys localization of the SA3-CAS(4,3) active orbitals yields charge-localized orbitals (denoted *i*, *p*, and *b*, see Supplementary Figure 2) across relevant geometries. These charge-localized orbitals were then used to express the six singlet configuration state functions (CSFs), yielding covalent and ionic subspaces (Supplementary Figure 3). Covalent CSFs are characterized by one doubly-occupied orbital and two singly-occupied orbital whereas ionic CSFs support two doubly-occupied orbitals and one empty orbital. Quasi-diabatic states are then obtained by a unitary block-diagonalization of the resulting configuration interaction Hamiltonian into covalent and ionic subspaces. The covalent-dominated diabatic states are labeled as $|X\rangle$, $X = I, P$ or B according to their respective doubly-occupied orbital. The Boys localization and construction of the Hamiltonian in the covalent/ionic CSF basis were performed with MolPro[4,5] whereas the α-CASSCF were obtained with TeraChem[6-9] and added manually to the adiabatic energies prior to block-diagonalization (recall that α-CASSCF does not affect the eigenstates).

Supplementary Methods 2: Cone sampling and photoisomerization committor

To investigate the origin of the improved photoreactivity of the trifluorinated chromophore, we performed so-called cone sampling and photoisomerization committor analysis of $TFHBDI^{-}$ following the procedure in our previous work on $HBDI^{-}$.[10] Specifically, we compared the photochemical outcome of ground-state dynamics starting from geometries sampled within the branching space of the respective MECI with three different velocity schemes: (i) minimum-energy paths, corresponding to quenching the kinetic energy after each infinitesimal time step; (ii) zero initial velocities; and (iii) random initial velocities. In scheme (iii), the atomic velocities were randomly sampled from a Gaussian distribution (zero mean and unity standard deviation) followed by mass-weighting and removal of translational and rotational motion. A uniform scaling was then applied to achieve an initial kinetic energy that equals the sum of the kinetic energy of the ground state ($HBDI^{-}$: 2.79 eV within a harmonic approximation) and the energy gap (~0.44 eV) between

the FC point and MECI-I$^+$ in HBDI$^-$. We used 50 random velocity initial conditions for each geometric displacement to estimate the committor surface for photoisomerization. The committor for each displacement was determined as the fraction of samples that reached the *E*-isomer before possibly undergoing non-statistical isomerization on S_0 back to the *Z*-isomer or vice versa (not observed within the considered 300 fs of S_0 dynamics). The initial geometries were sampled within the branching space of each MECI, as spanned by its characteristic gradient difference and derivative coupling vectors. The polar (0-360°) and radial coordinates defining the geometric displacements within the branching space were discretized in 18 (20° increments) and 4 steps (between 0.005 and 0.02 a_0), respectively. The ground state dynamics was performed at the same electronic-structure level of theory as the AIMS simulations (see Methods in the main text).

Supplementary Note 1: Validation of α-CASSCF against XMS-CASPT2

The nonadiabatic dynamics of TFHBDI$^-$ following photoexcitation to S_1 was modeled using AIMS with adiabatic energies, nuclear gradients and non-adiabatic couplings computed with the complete active space self-consistent field (CASSCF) implementation[11-13] in a development version of the graphical-processing-unit-accelerated TeraChem program.[6-8] To compare to our previous simulations on gas-phase HBDI$^-$,[10] we adopt the same computational setup. Specifically, we use the empirically-corrected α-CASSCF[14] method with an α-parameter of 0.64. This value was obtained for HBDI$^-$ by fitting to extended multistate multireference second-order perturbation theory (XMS-CASPT2) at the Franck-Condon (FC) point. As shown below, this value is transferable to TFHBDI$^-$. For MHBDI$^-$, a marginally smaller value (0.63) obtained a better fit at the FC point and was used in the three-state diabatic analysis. An active space consisting of four electrons in three orbitals (the bonding, non-bonding and anti-bonding methine bridge orbitals, see Supplementary Figure 2) with averaging over the three lowest singlet states and the 6-31G* basis set, i.e., α(0.64/0.63)-SA3-CASSCF(4,3)/6-31G* was used. The three-state averaging is necessary to provide a balanced description of the photoisomerization in the anionic HBDI$^-$ chromophore, permitting deactivation through both twisted modes.[1] Supplementary Figure 4 shows a comparison of relative energies for the SA3-XMS-CASPT2(4,3)/6-31G* (frozen core; level shift of 0.3 a.u.; SVP-jkfit density-fitting basis) reference and α(0.64)-SA3-CASSCF(4,3)/6-31G* levels at critical points for TFHBDI$^-$. Critical point geometries were optimized at their respective levels of theory. As seen, α(0.64)-SA3-CASSCF matches the XMS-CASPT2 reference at the FC point and

reproduces the relative energetic ordering of the different geometries. Consistent with the previous results for $HBDI^-$ (Figure S4 of Ref. 10), the energies of the twisted geometries are almost uniformly over-stabilized by ~0.2 eV. For $TFHBDI^-$, This leads to energy differences relative to the S_1-planar minimum for S_1-P and S_1-I of -0.23 eV and -0.69 eV, respectively, as compared with -0.03 and -0.56 eV at the XMS-CASPT2 level. Accordingly, progress along the P-torsional mode may be more accessible in the α-CASSCF dynamics than expected from the reference. It should be noted that the MECI-P geometry remains ~0.5 eV (~0.7 eV at XMS-CASPT2 level) above the FC point, limiting the access to the P-twisted intersection seam. Energies and selected geometric parameters for each level of theory are reported in Supplementary Tables 1-3 and 5-9, respectively.

Supplementary Note 2: Methoxy orientation in $MHBDI^-$

Supplementary Figure 6 shows a ground-state relaxed scan of the ϕ_{OCH3} dihedral angle (defined as $\angle H_3COC''C_3$) in $MHBDI^-$ at the α-CASSCF level. The ground-state minimum corresponds to an out-of-plane configuration with a more sp^3-hybridized methoxy group that points toward the phenolate O atom. There are two in-plane configurations where the methoxy group becomes increasingly sp^2-hybridized. Of these two configurations, only the one with the methoxy pointing away from the phenolate O atom represents a local minimum located 0.13 eV (0.09 eV at the XMS-CASPT2 level) above the out-of-plane minimum. Supplementary Tables 3/4 report the relative energies and 8/9 and 12/13 key geometric parameters for critical points at each of the two configurations (referred to as out-of-plane and in-plane methoxy orientation).

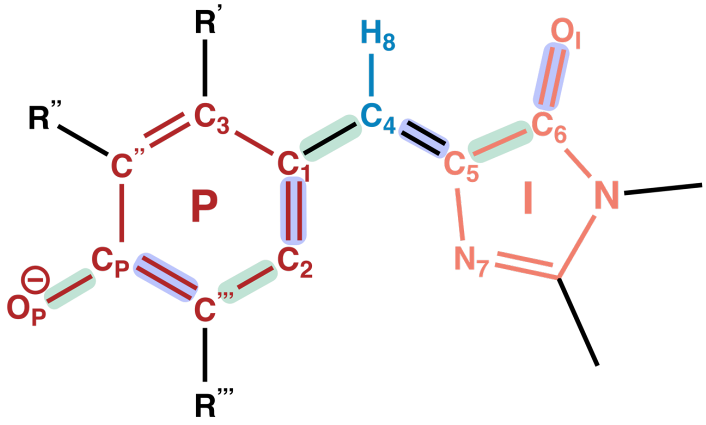

Definition of key geometric parameters

$$\theta_{\text{pyr}} = \arccos\left(\left(\mathbf{e}_{C_1\text{-}C_4} \times \mathbf{e}_{C_5\text{-}C_4}\right) \cdot \mathbf{e}_{H_8\text{-}C_4}\right) - \frac{\pi}{2}$$

$$\phi_I = \text{sgn}_I \cdot \arccos\left(\left(\mathbf{e}_{C_1\text{-}C_4} \times \mathbf{e}_{C_5\text{-}C_4}\right) \cdot \left(\mathbf{e}_{C_5\text{-}C_4} \times \mathbf{e}_{C_6\text{-}N_7}\right)\right)$$

$$\text{sgn}_I = \text{sgn}\left(\left(\left(\mathbf{e}_{C_1\text{-}C_4} \times \mathbf{e}_{C_5\text{-}C_4}\right) \times \left(\mathbf{e}_{C_5\text{-}C_4} \times \mathbf{e}_{C_6\text{-}N_7}\right)\right) \cdot \mathbf{e}_{C_5\text{-}C_4}\right)$$

$$\phi_P = \text{sgn}_P \cdot \arccos\left(\left(\mathbf{e}_{C_5\text{-}C_4} \times \mathbf{e}_{C_1\text{-}C_4}\right) \cdot \left(\mathbf{e}_{C_1\text{-}C_4} \times \mathbf{e}_{C_3\text{-}C_2}\right)\right)$$

$$\text{sgn}_P = \text{sgn}\left(\left(\left(\mathbf{e}_{C_5\text{-}C_4} \times \mathbf{e}_{C_1\text{-}C_4}\right) \times \left(\mathbf{e}_{C_1\text{-}C_4} \times \mathbf{e}_{C_3\text{-}C_2}\right)\right) \cdot \mathbf{e}_{C_1\text{-}C_4}\right)$$

Supplementary Figure 1. Definition of pyramidalization and dihedral angles in $HBDI^{-}$ and P-ring substituted derivatives ($MHBDI^{-}$: R'=R'''=H and R''=OCH_3; $TFHBDI^{-}$: R'=R''=R'''=F). Sign factors are given by the projection of the cross product of the normal vectors along the central bond. Note that the dihedral angles are defined with opposite handedness. With this definition of the pyramidalization angle, an idealized sp^2 C atom would give 0° while an idealized sp^3, as in methane, corresponds to 55°. The BLA coordinate used in Supplementary Figure 6 is defined as the difference between the sum of the double bonds (shaded blue boxes) and single bonds (shaded green boxes).

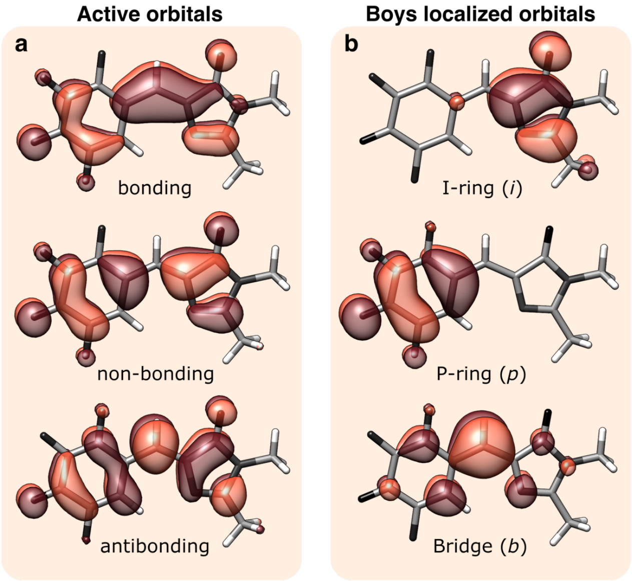

Supplementary Figure 2. Active-space orbitals. (a) The three orbitals constituting the active space for the SA3-α(0.64)-CASSCF and SA3-XMS-CASPT2 calculations, given at the FC geometry of $TFHBDI^{-}$. The active space orbitals for $MHBDI^{-}$ and $HBDI^{-}$ are of similar character (not shown). (b) Localized fragment orbitals on the I-ring (*i*), P-ring (*p*) and bridge (*b*) fragments as obtained by Boys localization. Isovalue: 0.03 a.u.

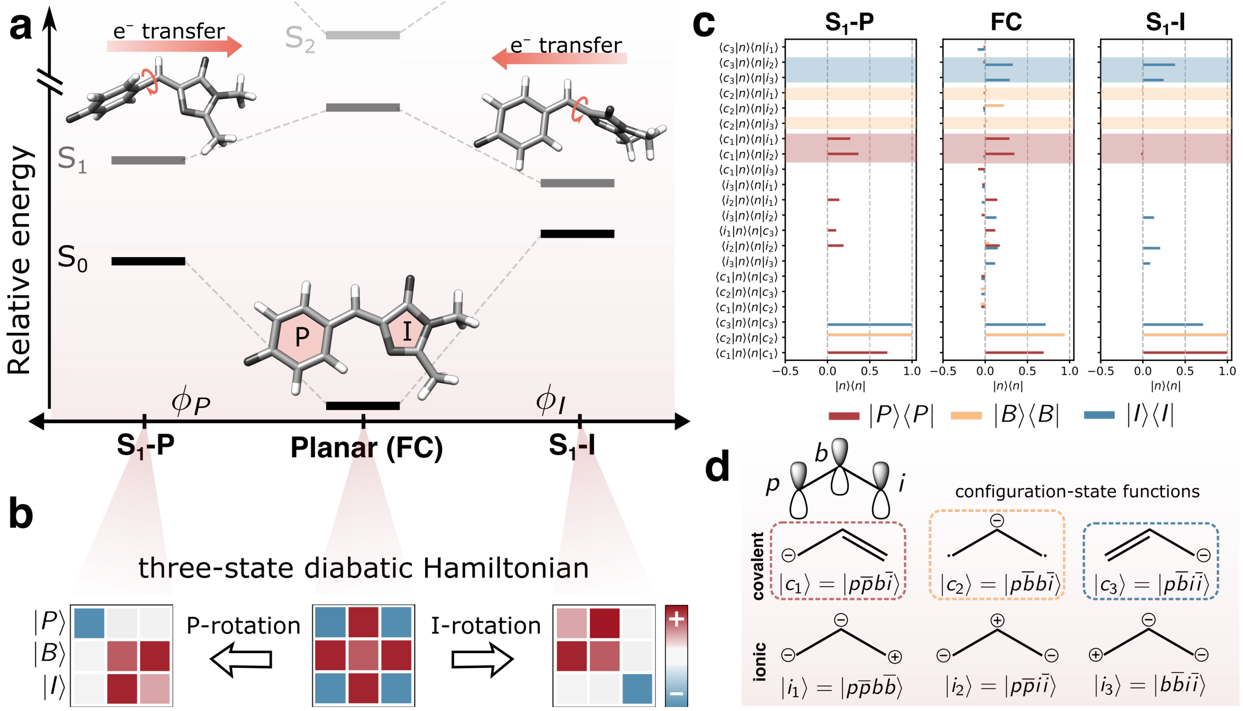

Supplementary Figure 3. Coupling between bridge-torsional motion and intramolecular charge-transfer character in HBDI⁻. (a) Torsional dependence of the S_0 and S_1 energies and the direction of intramolecular charge-transfer on S_1, computed at the α(0.64)-SA3-CASSCF(4,3)/6-31G* level. Underlying adiabatic state energies are reported in Supplementary Table 6. (b) Schematic representation of the three-state diabatic Hamiltonian and how it changes upon bridge-torsional deformations.[1] Displacement along the torsional coordinates leads to a block-diagonal form. The colored shadings indicate the relative sign and magnitude of the matrix elements. (c) Decomposition of the diabatic states in terms of the underlying configuration-state functions (CSFs), see (d). Within this orthonormalized charge-localized basis, bond formation is a consequence of coupling between the leading covalent configuration (to a given diabatic state) and its corresponding bond-polarizing ionic configurations. The color-coding indicates diabatic states, and the same-colored shaded areas highlights the bond-stabilizing ionic contributions for each diabatic state. (d) Schematic of the three fragment-localized orbitals (see also Supplementary Figure 2) together with the six singlet CSFs that can be generated by distributing four electrons in these three orbitals.

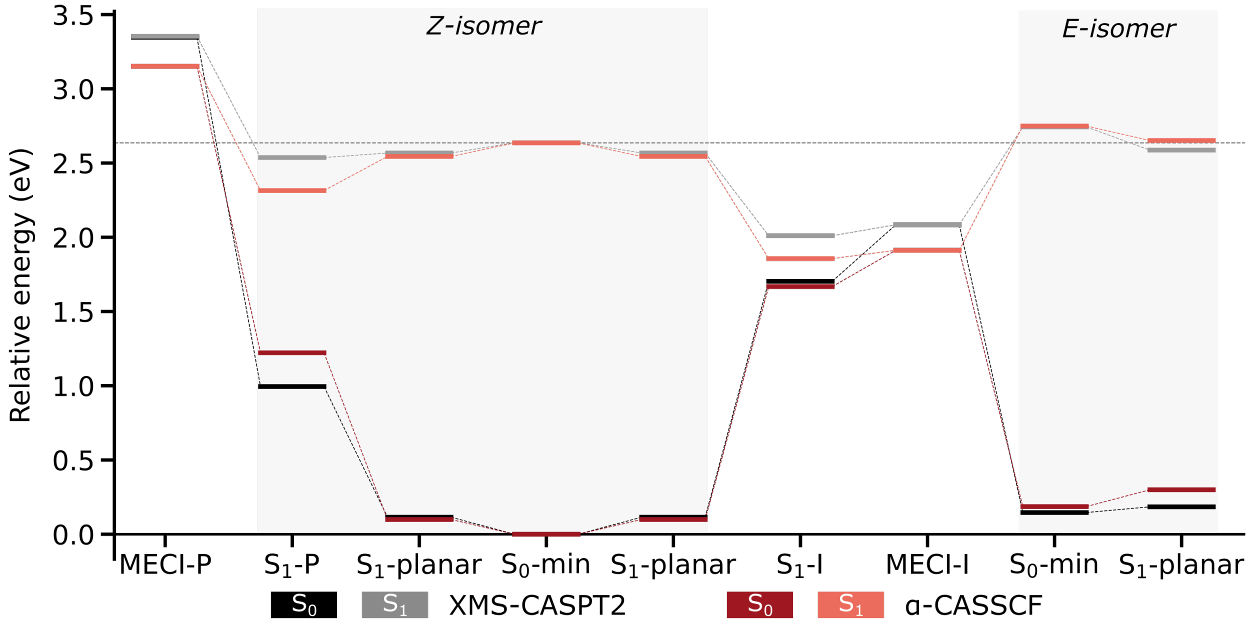

Supplementary Figure 4. Comparison of S_0 and S_1 potential energies for TFHBDI⁻ at important geometries obtained using SA3-α(0.64)-CASSCF(4,3)/6-31G* (red shades) and SA3-XMS-CASPT2/6-31G* (gray shades) at their respective levels of theory (tabulated energies provided in Supplementary Table 2). The α-parameter previously obtained for HDBI⁻ is also optimal for TFHBDI⁻. While α-CASSCF tends to over-stabilize twisted structures relative to planar structures, it correctly reproduces the relative ordering and that MECI-P lies above the FC point, while MECI-I lies below. Note that S_1-planar is not a true minimum at the α-CASSCF level of theory. The torsional barriers (eV) are shown above the curved dashed lines.

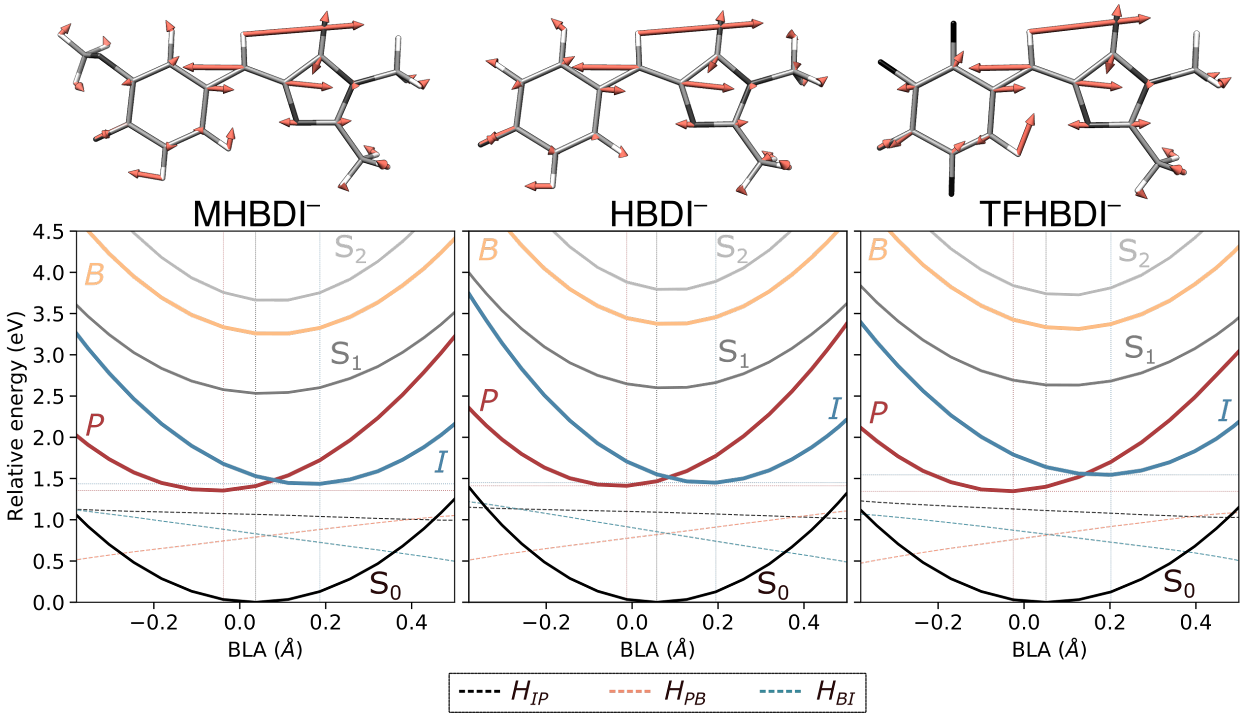

Supplementary Figure 5. Substituent effects from the perspective of the three-state diabatic model (see also Supplementary Figure 3). Adiabatic and diabatic state energies along a bond-length alternation (BLA, defined in Supplementary Figure 1) mode for each MHBDI^{-}, HBDI^{-} and TFHBDI^{-} (atomic displacement vectors smaller than 5% of the maximum are omitted for clarity). Both 2,3,5-trifluorination and 3-methoxylation increase the energy gap between the P and I diabatic states. However, the smaller couplings in MHBDI^{-} mean that the resulting adiabatic energy gap is red-shifted compared to HBDI^{-} (see Supplementary Table 5). Vertical lines indicate the BLA values at the minimum of the P and I diabatic states and S_0. Results were obtained at the α(0.63/0.64)-CASSCF(4,3)/6-31G* level.

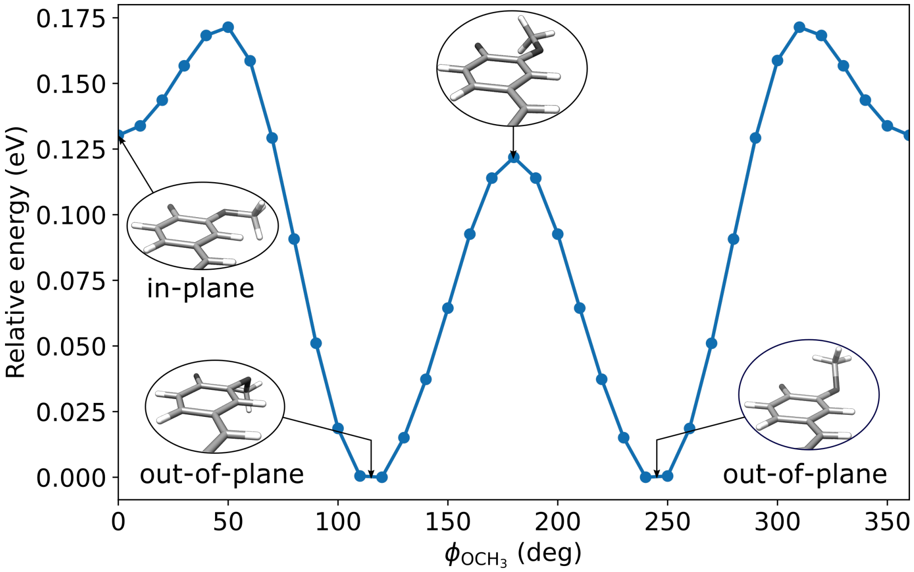

Supplementary Figure 6. Ground-state energy profile for a relaxed scan of methoxy dihedral angle ϕ_{OCH3} (defined as ∠$H_3COC''C_3$). The out-of-plane methoxy configuration is the global minimum while the in-plane configuration with the methoxy group pointing away from the phenolate O atom is a local minimum. The relaxed scan was performed at the α(0.63)-SA3-CASSCF(4,3)/6-31G* level of theory.

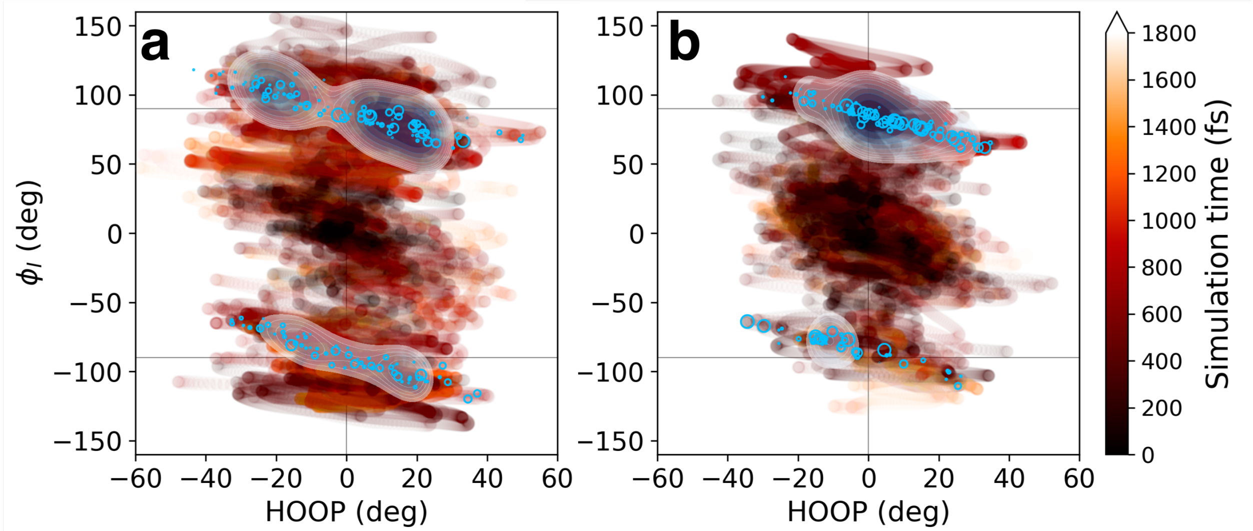

Supplementary Figure 7. Time evolution (see color code) of the centroids of the trajectory basis functions in the subspace spanned by the HOOP and I-torsional modes for (a) $HBDI^-$ and (b) $TFHBDI^-$. The faster HOOP mode initially follows the rotation direction of the I-dihedral, while the distribution is wider and centered around 0° when the I-torsion reaches ~90°. As indicated by the blue open circles, the non-adiabatic transition events occur at out-of-phase configurations. The areas of the circles are proportional to the absolute population transfer. (b) The blue contours show the associated absolute population transfer (i.e., each spawn is weighted by the population transferred and convolved with a Gaussian function) and highlights the bimodal and unimodal distributions in $HBDI^-$ and $TFHBDI^-$, respectively. The somewhat asymmetric spawning distributions relative to the I-torsion direction is likely a consequence of the relatively small number of initial conditions used in this work since the underlying potential energy profile is symmetric.

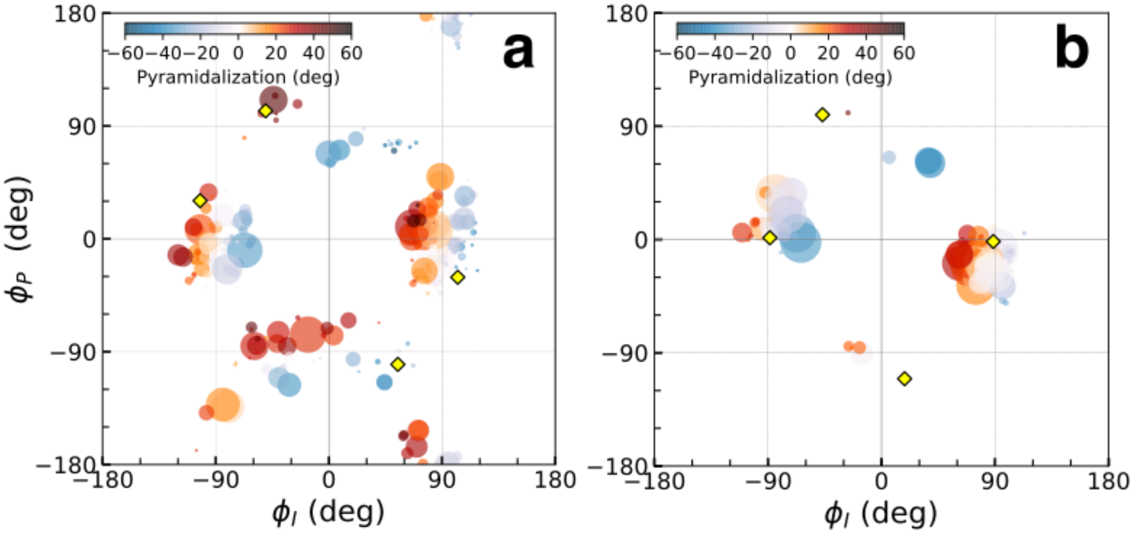

Supplementary Figure 8. Distribution of (ϕ_I, ϕ_P)-dihedrals at the spawning geometries. The radius and color of each circle represent absolute population transfer and extent of bridge pyramidalization, respectively. The absolute population transfer is defined as the total population gained by the child TBF from the beginning of the coupled propagation until the gain drops below a threshold value of 10^{-4}. Efficient population transfer is associated with significant pyramidalization of the methine bridge. Yellow diamonds indicate the location of MECIs.

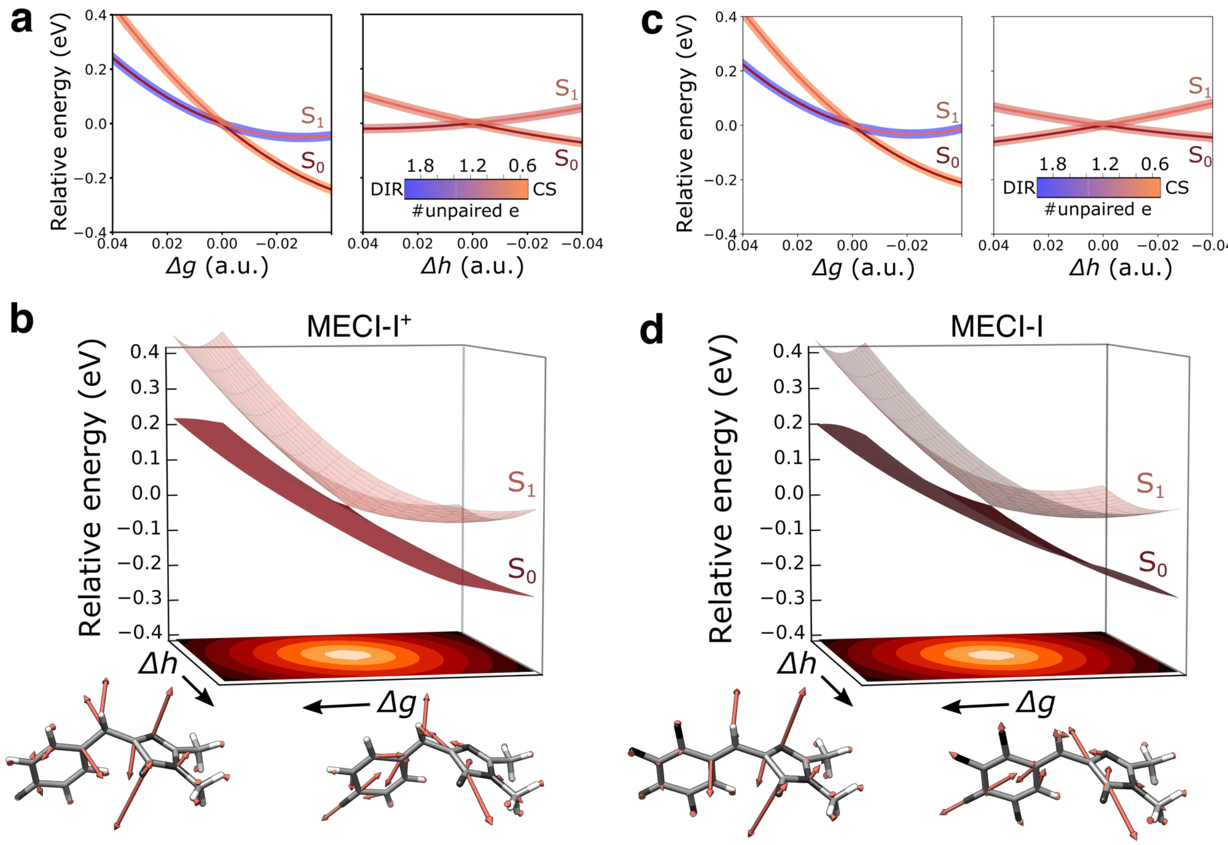

Supplementary Figure 9. Local topographies of the MECI-Is of HBDI$^-$ and TFHBDI$^-$. (a) S_1 and S_0 potential energy cuts for MECI-I$^+$ along the direction of the *g*- and *h*-vectors (defined according to Yarkony's procedure[15] and imposing that $\Delta_{gh} \geq 0$ and $\theta_s \in \left[0, \frac{\pi}{2}\right]$[16]) with line shading indicating the electronic character of the adiabatic state in terms of effective number of unpaired electrons:[17] a value close to two indicates a diradical (DIR) character which is shaded in blue, while a value close to zero indicates the closed-shell (CS) charge-transfer electronic configuration shaded in orange. (b) Cone plot for MECI-I in TFHBDI$^-$ for displacements of ±0.04 a.u. (c) and (d) are the corresponding plots for MECI-P$^+$. S_1 and S_0 energies are reported with respect to that of the respective MECI and the energy gap is shown as a contour plot. Intersection parameters, as defined in Ref. 16, are provided in Supplementary Table 15. Both MECIs are sloped in the direction of the gradient vector while peaked along the derivative coupling vector. The arrows on the molecules represent the *g* and *h* unit-vectors (ranges extend from ±0.04 a.u. where black arrows indicate positive displacement direction). For MECI-I$^+$ in HBDI$^-$, the gradient difference corresponds to a combination of bond-length alternation and HOOP motion, while the non-adiabatic coupling vector is dominated by a linearized torsional motion along ϕ_I. In TFHBDI$^-$, HOOP motion does not substantially contribute to the *g*-vector.

Supplementary Table 1. SA3-α(0.64)-CASSCF(4,3)/6-31G* and SA3-XMS-CASPT2(4,3)/6-31G* energies (eV) for critical points of $HBDI^-$ optimized at their respective level of theory. Energies are reported relative to the ground state energy at the corresponding FC point.

	S_0-min	S_1-planar	S_1-I	S_1-P	MECI-I^+	MECI-$I2^+$	MECI-P^+	S_0 (E)	S_1-planar (E)
					α-CASSCF				
S_0	0.000	0.094	1.499	1.260	2.156	2.163	2.675	0.154	0.257
S_1	2.600	2.514[a]	1.941	2.140	2.156	2.163	2.675	2.736	2.644
					XMS-CASPT2[b]				
S_0	0.000	0.095	1.477	1.087	2.459	2.491	2.922	0.138	0.240
S_1	2.608	2.548	2.239	2.414	2.459	2.492	2.922	2.736	2.651

[a] A first-order saddle point on S_1 rather than a minimum (see Ref. [10]).
[b] Small differences to Supplementary Table 1 in Ref. [10] due to using the Cartesian instead of spherical basis representation.

Supplementary Table 2. SA3-α(0.64)-CASSCF(4,3)/6-31G* and SA3-XMS-CASPT2(4,3)/6-31G* energies (eV) for critical points of $TFHBDI^-$ optimized at their respective level of theory. Energies are reported relative to the ground state energy at the corresponding FC point.

	S_0-min	S_1-planar	S_1-I	S_1-P	MECI-I	MECI-P^+	S_0 (E)	S_1-planar (E)
					α-CASSCF			
S_0	0.000	0.099	1.668	1.222	1.912	3.152	0.186	0.300
S_1	2.636	2.544	1.856	2.315	1.912	3.152	2.750	2.652
					XMS-CASPT2			
S_0	0.000	0.115	1.703	0.995	2.084	3.347	0.146	0.184
S_1	2.637	2.568	2.011	2.537	2.084	3.347	2.743	2.587

Supplementary Table 3. SA3-α(0.63)-CASSCF(4,3)/6-31G* and SA3-XMS-CASPT2(4,3)/6-31G* energies (eV) for critical points of $MHBDI^-$ (out-of-plane methoxy orientation) optimized at their respective level of theory. Energies are reported relative to the ground state energy at the corresponding FC point.

	S_0-min	S_1-planar	S_1-I	S_1-P	MECI-I^+	MECI-P^+	S_0 (E)	S_1-planar (E)
					α-CASSCF			
S_0	0.000	0.090	1.509	1.240	2.069	2.666	0.149	0.583
S_1	2.531	2.448	1.887	2.119	2.069	2.666	2.661	2.825
					XMS-CASPT2			
S_0	0.000	0.171	1.527	0.996	2.341	2.780	0.134	0.284
S_1	2.536	2.493	2.157	2.330	2.341	2.780	2.657	2.784

Supplementary Table 4. SA3-α(0.63)-CASSCF(4,3)/6-31G* and SA3-XMS-CASPT2(4,3)/6-31G* energies (eV) for critical points of MHBDI⁻ (in-plane methoxy orientation) optimized at their respective level of theory. Energies are reported relative to the ground state energy at the corresponding FC point.

	S_0-min	S_1-planar	S_1-I	S_1-P	MECI-I$^+$	MECI-P$^+$	S_0 (*E*)	S_1-planar (*E*)
				α-CASSCF				
S_0	0.000	0.093	1.447	1.285	2.139	2.403	0.154	0.251
S_1	2.504	2.420	1.899	2.018	2.139	2.403	2.646	2.556
				XMS-CASPT2				
S_0	0.000	0.104	1.426	1.140	2.444	2.585	0.150	0.196
S_1	2.521	2.431	2.212	2.225	2.444	2.585	2.674	2.532

Supplementary Table 5. Substituent effects on the vertical excitation energies of HBDI⁻. Results were obtained with SA3-XMS-CASPT2(4,3)/6-31G* at FC geometries computed at the same level of theory. The directionality of the shift for MHBDI⁻ is consistent with that reported for the symmetric 3,5-dimethoxy derivative whereas the blue-shift for TFHBDI⁻ is opposite of that for the 3,5-difluoro derivative (red-shift of <0.1 eV).[18] Our calculations on the difluoro analogue reproduces this substitution pattern (i.e., 3-methoxy<3,5-difluoro<HBDI⁻<2,3,5-trifluoro).

System	ΔE (eV)
HBDI⁻	2.608
3-methoxy (MHBDI⁻)	2.536
3,5-difluoro	2.581
2,3,5-trifluoro (TFHBDI⁻)	2.637

Supplementary Table 6. Selected geometric parameters at critical points for $HBDI^{-}$ optimized at the α(0.64)-SA3-CASSCF(4,3)/6-31G* level of theory. Distances are given in Ångström and angles, dihedrals and pyramidalization in degrees with definitions provided in Supplementary Figure 1.

	S_0-min	S_1-planar[a]	S_1-I	S_1-P	MECI-I^{+}	MECI-P^{+}	S_0-min (*E*)	S_1-planar (*E*)
R_P	1.411	1.445	1.414	1.471	1.447	1.485	1.411	1.442
R_I	1.395	1.435	1.457	1.410	1.469	1.462	1.402	1.447
C-O_P	1.228	1.230	1.235	1.219	1.244	1.202	1.227	1.230
C-O_I	1.218	1.222	1.206	1.229	1.190	1.251	1.219	1.224
C_5-N_7	1.390	1.368	1.376	1.384	1.358	1.400	1.399	1.374
C_5-C_6	1.442	1.442	1.460	1.429	1.496	1.391	1.444	1.444
$\angle C_1C_4C_5$	131.5	128.1	124.8	123.5	118.0	108.3	135.1	131.9
ϕ_I	0.0	0.2	88.2	0.0	102.5	-50.3	180.0	179.5
ϕ_P	0.0	-0.2	-1.2	-89.6	-30.9	101.0	0.0	-0.2
θ_{pyr}	0.0	-0.1	0.3	-0.1	-29.6	48.3	0.0	0.1

[a] A first-order saddle point on S_1 rather than a minimum

Supplementary Table 7. Selected geometric parameters at critical points for $TFHBDI^{-}$ optimized at the α(0.64)-SA3-CASSCF(4,3)/6-31G* level of theory. Distances are given in Ångström and angles, dihedrals and pyramidalization in degrees with definitions provided in Supplementary Figure 1.

	S_0-min	S_1-planar[a]	S_1-I	S_1-P	MECI-I^{+}	MECI-P^{+}	S_0-min (*E*)	S_1-planar (*E*)
R_P	1.411	1.432	1.408	1.466	1.420	1.480	1.412	1.429
R_I	1.389	1.439	1.458	1.408	1.463	1.471	1.397	1.452
C-O_P	1.220	1.221	1.226	1.210	1.236	1.188	1.220	1.221
C-O_I	1.214	1.221	1.205	1.227	1.192	1.252	1.216	1.225
C_5-N_7	1.391	1.366	1.373	1.383	1.361	1.407	1.398	1.370
C_5-C_6	1.447	1.439	1.459	1.429	1.487	1.386	1.449	1.441
$\angle C_1C_4C_5$	130.1	126.8	123.8	122.3	122.6	102.7	134.0	130.8
ϕ_I	0.3	0.4	88.7	-0.2	88.6	-46.7	180.0	180.0
ϕ_P	-0.2	-0.2	-0.7	-89.6	-1.6	98.8	-0.1	-0.1
θ_{pyr}	-0.1	-0.2	0.2	-0.8	0.4	54.2	-0.1	-0.1

[a] A first-order saddle point on S_1 rather than a minimum

Supplementary Table 8. Selected geometric parameters at critical points for MHBDI$^-$ (out-of-plane methoxy orientation) optimized at the α(0.63)-SA3-CASSCF(4,3)/6-31G* level of theory. Distances are given in Ångström and angles, dihedrals and pyramidalization in degrees with definitions provided in Supplementary Figure 1.

	S_0-min	S_1-planar[a]	S_1-I	S_1-P	MECI-I$^+$	MECI-P$^+$	S_0-min (*E*)	S_1-planar (*E*)
R_P	1.412	1.445	1.412	1.471	1.439	1.488	1.412	1.466
R_I	1.395	1.432	1.458	1.410	1.468	1.462	1.402	1.394
C-O_P	1.230	1.232	1.237	1.220	1.249	1.201	1.229	1.242
C-O_I	1.217	1.222	1.206	1.229	1.190	1.251	1.219	1.226
C_5-N_7	1.390	1.369	1.375	1.384	1.360	1.400	1.398	1.400
C_5-C_6	1.443	1.442	1.460	1.429	1.495	1.391	1.445	1.445
$\angle C_1C_4C_5$	131.3	127.9	124.7	123.3	119.2	108.2	135.0	132.0
ϕ_{OCH3}[b]	115.5	117.5	117.1	114.6	118.7	107.9	115.7	154.9
ϕ_I	-0.1	-0.2	88.2	0.1	101.0	-49.7	179.9	-179.3
ϕ_P	0.3	0.4	-0.1	90.3	-25.5	103.2	0.3	-0.7
θ_{pyr}	0.0	0.0	0.4	0.1	-25.3	48.4	0.0	-0.3

[a] A first-order saddle point on S_1 rather than a minimum.
[b] Orientation of the methoxy group defined by the dihedral angle $\angle H_3COC''C_3$.

Supplementary Table 9. Selected geometric parameters at critical points for MHBDI$^-$ (in-plane methoxy orientation) optimized at the α(0.63)-SA3-CASSCF(4,3)/6-31G* level of theory. Distances are given in Ångström and angles, dihedrals and pyramidalization in degrees with definitions provided in Supplementary Figure 1.

	S_0-min	S_1-planar[a]	S_1-I	S_1-P	MECI-I$^+$	MECI-P$^+$	S_0-min (*E*)	S_1-planar (*E*)
R_P	1.410	1.448	1.411	1.473	1.442	1.493	1.410	1.482
R_I	1.396	1.430	1.457	1.409	1.469	1.455	1.403	1.392
C-O_P	1.224	1.226	1.231	1.216	1.242	1.202	1.223	1.232
C-O_I	1.218	1.222	1.206	1.230	1.189	1.249	1.220	1.224
C_5-N_7	1.390	1.370	1.376	1.384	1.357	1.395	1.399	1.396
C_5-C_6	1.442	1.443	1.460	1.429	1.499	1.395	1.443	1.450
$\angle C_1C_4C_5$	131.5	127.9	124.6	123.6	117.2	112.3	135.1	132.2
ϕ_{OCH3}[b]	0.0	-0.1	-0.7	2.0	0.4	1.8	-0.1	-0.4
ϕ_I	0.0	1.0	88.0	-0.5	102.3	-47.9	-180.0	-178.5
ϕ_P	0.0	-0.6	-1.1	91.2	-29.3	106.5	-0.1	-2.2
θ_{pyr}	0.0	-0.4	0.3	0.6	-30.6	44.1	0.0	-0.7

[a] A first-order saddle point on S_1 rather than a minimum.
[b] Orientation of the methoxy group defined by the dihedral angle $\angle H_3COC''C_3$.

Supplementary Table 10. Selected geometric parameters at critical points for $HBDI^{-}$ optimized at the SA3-XMS-CASPT2(4,3)/6-31G*[a] level of theory. Distances are given in Ångström and angles, dihedrals and pyramidalization in degrees with definitions provided in Supplementary Figure 1.

	S_0-min	S_1-planar	S_1-I	S_1-P	MECI-I^+	MECI-P^+	S_0-min (E)	S_1-planar (E)
R_P	1.407	1.447	1.407	1.470	1.457	1.472	1.404	1.447
R_I	1.390	1.414	1.456	1.401	1.461	1.484	1.398	1.423
C-O_P	1.262	1.277	1.277	1.271	1.284	1.257	1.262	1.278
C-O_I	1.245	1.252	1.238	1.262	1.221	1.281	1.249	1.257
C_5-N_7	1.398	1.385	1.356	1.387	1.333	1.391	1.404	1.391
C_5-C_6	1.457	1.460	1.468	1.449	1.504	1.415	1.458	1.465
$\angle C_1C_4C_5$	131.6	127.8	122.7	121.1	113.9	103.3	135.0	132.0
ϕ_I	0.0	0.1	88.6	0.0	104.6	-53.7	179.9	178.8
ϕ_P	0.1	-0.1	-1.6	-90.2	-30.6	96.7	0.0	-0.4
θ_{pyr}	0.0	0.0	-1.1	-0.1	-38.5	54.3	0.0	0.4

[a] Small differences to Table S4 in Ref. 10 due to using the Cartesian instead of spherical one-electron basis.

Supplementary Table 11. Selected geometric parameters at critical points for $TFHBDI^{-}$ optimized at the SA3-XMS-CASPT2(4,3)/6-31G* level of theory. Distances are given in Ångström and angles, dihedrals and pyramidalization in degrees with definitions provided in Supplementary Figure 1.

	S_0-min	S_1-planar	S_1-I	S_1-P	MECI-I^+	MECI-P^+	S_0-min (E)	S_1-planar (E)
R_P	1.411	1.442	1.400	1.464	1.417	1.493	1.409	1.439
R_I	1.383	1.416	1.456	1.400	1.457	1.470	1.390	1.428
C-O_P	1.257	1.267	1.271	1.261	1.278	1.414	1.256	1.267
C-O_I	1.241	1.253	1.238	1.259	1.228	1.276	1.246	1.260
C_5-N_7	1.398	1.383	1.354	1.388	1.340	1.383	1.405	1.388
C_5-C_6	1.462	1.456	1.467	1.447	1.487	1.425	1.463	1.459
$\angle C_1C_4C_5$	129.9	126.2	121.8	119.1	119.3	105.4	133.7	130.3
ϕ_I	0.0	0.2	88.8	5.6	98.1	-39.4	179.9	-179.4
ϕ_P	0.1	-0.5	-1.1	-93.5	-13.7	105.5	-0.1	-0.6
θ_{pyr}	0.0	-0.2	-0.4	-10.3	-17.7	54.0	0.0	-0.2

Supplementary Table 12. Selected geometric parameters at critical points for MHBDI$^-$ (out-of-plane methoxy orientation) optimized at the SA3-XMS-CASPT2(4,3)/6-31G* level of theory. Distances are given in Ångström and angles, dihedrals and pyramidalization in degrees with definitions provided in Supplementary Figure 1. Note that the methoxy group upon optimization of MECI-P$^+$ ends up being essentially in plane with the ring.

	S_0-min	S_1-planar	S_1-I	S_1-P	MECI-I$^+$	MECI-P$^+$	S_0-min (*E*)	S_1-planar (*E*)
R_P	1.410	1.451	1.405	1.470	1.445	1.492	1.408	1.458
R_I	1.387	1.407	1.457	1.401	1.461	1.468	1.396	1.408
C-O_P	1.265	1.278	1.280	1.273	1.290	1.278	1.265	1.282
C-O_I	1.244	1.253	1.238	1.262	1.223	1.280	1.248	1.256
C_5-N_7	1.398	1.389	1.356	1.387	1.335	1.388	1.405	1.403
C_5-C_6	1.459	1.461	1.468	1.449	1.498	1.420	1.460	1.461
$\angle C_1C_4C_5$	131.4	127.6	123.4	120.6	115.8	107.7	134.9	132.2
ϕ_{OCH3}[a]	122.2	133.4	123.9	132.7	126.1	178.8	122.5	157.7
ϕ_I	-0.1	-3.1	87.3	-0.2	103.2	-43.4	180.0	-178.0
ϕ_P	0.4	2.0	0.7	90.4	-25.3	106.6	0.5	-2.6
θ_{pyr}	0.1	1.9	0.8	-0.6	-32.9	49.7	0.1	-1.0

[a] Orientation of the methoxy group defined by the dihedral angle $\angle H_3COC''C_3$.

Supplementary Table 13. Selected geometric parameters at critical points for MHBDI$^-$ (in-plane methoxy orientation) optimized at the SA3-XMS-CASPT2(4,3)/6-31G* level of theory. Distances are given in Ångström and angles, dihedrals and pyramidalization in degrees with definitions provided in Supplementary Figure 1.

	S_0-min	S_1-planar	S_1-I	S_1-P	MECI-I$^+$	MECI-P$^+$	S_0-min (*E*)	S_1-planar (*E*)
R_P	1.406	1.455	1.402	1.471	1.453	1.488	1.403	1.452
R_I	1.391	1.407	1.455	1.401	1.460	1.467	1.400	1.417
C-O_P	1.259	1.270	1.275	1.269	1.283	1.271	1.258	1.271
C-O_I	1.246	1.252	1.238	1.262	1.221	1.280	1.250	1.256
C_5-N_7	1.397	1.388	1.357	1.387	1.332	1.387	1.404	1.394
C_5-C_6	1.456	1.462	1.468	1.449	1.508	1.420	1.456	1.466
$\angle C_1C_4C_5$	131.6	127.6	123.5	121.3	113.4	110.1	134.9	131.9
ϕ_{OCH3}[a]	0.1	0.2	-0.3	2.7	2.5	3.1	-0.1	-0.1
ϕ_I	0.1	0.1	87.7	0.6	104.2	-47.1	-179.9	-178.2
ϕ_P	0.1	0.4	-0.3	92.9	-29.3	107.2	0.0	-2.1
θ_{pyr}	0.0	0.1	-0.1	-0.4	-39.4	48.6	0.0	-1.1

[a] Orientation of the methoxy group defined by the dihedral angle $\angle H_3COC''C_3$.

Supplementary Table 14. Mulliken charges at important geometries for the ground and first excited state of $HBDI^{-}$, $TFHBDI^{-}$ and $MHBDI^{-}$ obtained at the α(0.64/0.63)-SA3-CASSCF(4,3)/6-31G* level of theory.

		S_0-min		S_1-planar		S_1-I		S_1-P	
		S_0	S_1	S_0	S_1	S_0	S_1	S_0	S_1
I-ring	$HBDI^{-}$	-0.74	-0.71	-0.74	-0.73	-1.26	-0.34	-0.39	-0.86
	$TFHBDI^{-}$	-0.69	-0.70	-0.71	-0.73	-1.26	-0.34	-0.36	-0.83
	$MHBDI^{-}$	-0.73	-0.71	-0.74	-0.73	-1.26	-0.34	-0.38	-0.86
P-ring	$HBDI^{-}$	-0.57	-0.48	-0.58	-0.50	-0.20	-0.69	-0.99	-0.05
	$TFHBDI^{-}$	-0.67	-0.55	-0.66	-0.56	-0.28	-0.77	-1.06	-0.13
	$MHBDI^{-}$	-0.57	-0.49	-0.58	-0.51	-0.21	-0.70	-1.00	-0.06
Bridge	$HBDI^{-}$	0.07	-0.03	0.08	-0.01	0.23	-0.21	0.16	-0.30
	$TFHBDI^{-}$	0.10	0.01	0.12	0.05	0.29	-0.15	0.18	-0.27
	$MHBDI^{-}$	0.07	-0.03	0.09	0.00	0.23	-0.20	0.16	-0.29
Methyl	$HBDI^{-}$	0.23	0.23	0.24	0.23	0.23	0.24	0.22	0.21
	$TFHBDI^{-}$	0.25	0.24	0.25	0.24	0.24	0.26	0.23	0.22
	$MHBDI^{-}$	0.24	0.23	0.24	0.23	0.23	0.24	0.22	0.21
O_P	$HBDI^{-}$	-0.73	-0.72	-0.78	-0.76	-0.68	-0.75	-0.75	-0.65
	$TFHBDI^{-}$	-0.69	-0.68	-0.68	-0.68	-0.62	-0.71	-0.72	-0.60
	$MHBDI^{-}$	-0.72	-0.72	-0.73	-0.72	-0.68	-0.75	-0.75	-0.64
O_I	$HBDI^{-}$	-0.71	-0.71	-0.73	-0.73	-0.74	-0.62	-0.67	-0.75
	$TFHBDI^{-}$	-0.69	-0.69	-0.70	-0.70	-0.74	-0.62	-0.66	-0.74
	$MHBDI^{-}$	-0.70	-0.70	-0.71	-0.71	-0.74	-0.62	-0.67	-0.75

Supplementary Table 15. Intersection parameters[a] for the MECI-Is in $HBDI^{-}$ and $TFHBDI^{-}$ at the α(0.64)-SA3-CASSCF(4,3)/6-31G* level of theory.

System	Type	ΔE (eV)	δ_{gh} (a.u.)	Δ_{gh} (a.u.)	σ	θ_s (deg)
$HBDI^{-}$	MECI-I^{+}	2.156	0.0400	0.4184	2.9221	5.9
	MECI-$I2^{+}$	2.163	0.0411	0.5490	3.2166	1.2
$TFHBDI^{-}$	MECI-I	1.912	0.0403	0.4068	2.5900	2.0

[a] ΔE: energy with respect to the ground-state energy at the relevant FC point, δ_{gh}: pitch, Δ_{gh}: asymmetry, σ, θ_s: relative tilt and tilt direction, as defined in Ref. [16].

Supplementary Table 16. Elements of the covalent subspace of the block effective Hamiltonian at representative geometries of $HBDI^-$ and derivatives (TF: $TFHBDI^-$) and (M: $MHBDI^-$) as obtained at the α(0.64/0.63)-CASSCF(4,3)/6-31G* level of theory. Energies are in kcal/mol and relative to the mean of the diagonal elements at the S_1-I geometry of the given system.

Elmt.\Geom	S_0 (*Z*)	S_1-I	S_1-P	S_0 (*Z*) (TF)	S_1-I (TF)	S_1-P (TF)	S_0 (*Z*) (M)	S_1-I (M)	S_1-P (M)
$\langle P \| H_{eff} \| P \rangle$	-27.8	9.2	-32.6	-29.4	6.9	-33.4	-27.7	8.8	-31.6
$\langle B \| H_{eff} \| B \rangle$	16.2	17.9	24.2	15.3	16.2	26.8	15.0	16.6	24.7
$\langle I \| H_{eff} \| I \rangle$	-25.9	-27.0	16.1	-23.8	-23.2	21.1	-25.0	-25.4	16.7
$\langle P \| H_{eff} \| B \rangle$	18.9	30.1	0.0	18.7	30.0	0.1	18.2	29.1	0.0
$\langle B \| H_{eff} \| I \rangle$	19.7	0.0	32.2	19.1	-0.0	32.0	19.2	0.1	31.7
$\langle P \| H_{eff} \| I \rangle$	-25.2	0.4	0.0	-25.7	-0.2	-0.3	-24.5	0.3	0.1

Supplementary Table 17. Elements of the covalent subspace of the block effective Hamiltonian at representative geometries of $HBDI^-$ and derivatives (TF: $TFHBDI^-$) and (M: $MHBDI^-$) as obtained at the α(0.64/0.63)-CASSCF(4,3)/6-31G* level of theory. Energies are in kcal/mol and relative to the mean of the diagonal elements at the S_1-I geometry of the given system.

Elmt.\Geom	MECI-I^+	MECI-$I2^+$	MECI-P^+	MECI-I^+ (TF)	MECI-P^+ (TF)	MECI-I^+ (M)	MECI-P^+ (M)
$\langle P \| H_{eff} \| P \rangle$	8.7	9.9	0.2	6.2	11.0	8.4	1.4
$\langle B \| H_{eff} \| B \rangle$	21.1	21.8	29.1	17.7	39.7	19.9	30.3
$\langle I \| H_{eff} \| I \rangle$	-11.9	-11.8	17.2	-17.6	33.6	-12.4	18.5
$\langle P \| H_{eff} \| B \rangle$	26.1	26.9	-1.9	28.9	0.6	26.0	-1.8
$\langle B \| H_{eff} \| I \rangle$	-0.3	-0.1	-22.3	0.0	-25.5	-0.1	-22.3
$\langle P \| H_{eff} \| I \rangle$	-0.2	-0.1	1.5	0.0	-0.5	-0.1	1.3

Supplementary References

1 Olsen, S. & McKenzie, R. H. A diabatic three-state representation of photoisomerization in the green fluorescent protein chromophore. *J. Chem. Phys.* **130**, 184302 (2009). https://doi.org:10.1063/1.3121324

2 Cederbaum, L. S., Schirmer, J. & Meyer, H. D. Block diagonalisation of Hermitian matrices. *J. Phys. A: Math. Gen.* **22**, 2427-2439 (1989). https://doi.org:10.1088/0305-4470/22/13/035

3 Pacher, T., Cederbaum, L. S. & Köppel, H. Approximately diabatic states from block diagonalization of the electronic Hamiltonian. *J. Chem. Phys.* **89**, 7367-7381 (1988). https://doi.org:10.1063/1.455268

4 H.-J. Werner, P. J. K., G. Knizia, F. R. Manby, M. Schütz, P. Celani, W. Györffy, D. Kats, T. Korona, R. Lindh, A. Mitrushenkov, G. Rauhut, K. R. Shamasundar, T. B. Adler, R. D. Amos, A. Bernhardsson, A. Berning, D. L.Cooper, M. J. O. Deegan, A. J. Dobbyn, F. Eckert, E. Goll, C. Hampel, A. Hesselmann, G. Het-zer, T. Hrenar, G. Jansen, C. Köppl, Y. Liu, A. W. Lloyd, R. A. Mata, A. J. May, S. J. McNi-cholas, W. Meyer, M. E. Mura, A. Nicklass, D. P. O'Neill, P. Palmieri, D. Peng, K. Pflüger,R. Pitzer, M. Reiher, T. Shiozaki, H. Stoll, A. J. Stone, R. Tarroni, T. Thorsteinsson, and M. Wang. *MOLPRO, version 2015.1, a package of ab initio programs, see http://www.molpro.net.*, 2015).

5 Werner, H.-J., Knowles, P. J., Knizia, G., Manby, F. R. & Schütz, M. Molpro: a general-purpose quantum chemistry program package. *WIREs Computational Molecular Science* **2**, 242-253 (2012). https://doi.org:10.1002/wcms.82

6 Ufimtsev, I. S. & Martinez, T. J. Quantum chemistry on graphical processing units. 1. Strategies for two-electron integral evaluation. *J. Chem. Theory Comput.* **4**, 222-231 (2008).

7 Ufimtsev, I. S. & Martinez, T. J. Quantum chemistry on graphical processing units. 2. Direct self-consistent-field implementation. *J. Chem. Theory Comput.* **5**, 1004-1015 (2009).

8 Ufimtsev, I. S. & Martinez, T. J. Quantum chemistry on graphical processing units. 3. Analytical energy gradients, geometry optimization, and first principles molecular dynamics. *J. Chem. Theory Comput.* **5**, 2619-2628 (2009).

9 Seritan, S. *et al.* TeraChem: Accelerating electronic structure and ab initio molecular dynamics with graphical processing units. *J. Chem. Phys.* **152**, 224110 (2020). https://doi.org:10.1063/5.0007615

10 List, N. H., Jones, C. M. & Martínez, T. J. Internal conversion of the anionic GFP chromophore: in and out of the I-twisted S1/S0 conical intersection seam. *Chemical Science* **13**, 373-385 (2022). https://doi.org:10.1039/D1SC05849E

11 Snyder, J. W., Jr., Fales, B. S., Hohenstein, E. G., Levine, B. G. & Martinez, T. J. A direct-compatible formulation of the coupled perturbed complete active space self-consistent field equations on graphical processing units. *J. Chem. Phys.* **146**, 174113 (2017). https://doi.org:10.1063/1.4979844

12 Snyder, J. W., Jr., Curchod, B. F. & Martinez, T. J. GPU-Accelerated State-Averaged Complete Active Space Self-Consistent Field Interfaced with Ab Initio Multiple Spawning Unravels the Photodynamics of Provitamin D3. *J. Phys. Chem. Lett.* **7**, 2444-2449 (2016). https://doi.org:10.1021/acs.jpclett.6b00970

13 Snyder Jr, J. W., Hohenstein, E. G., Luehr, N. & Martínez, T. J. An atomic orbital-based formulation of analytical gradients and nonadiabatic coupling vector elements for the state-

averaged complete active space self-consistent field method on graphical processing units. *The Journal of chemical physics* **143**, 154107 (2015).

14 Snyder, J. W., Jr., Parrish, R. M. & Martinez, T. J. alpha-CASSCF: An Efficient, Empirical Correction for SA-CASSCF To Closely Approximate MS-CASPT2 Potential Energy Surfaces. *J. Phys. Chem. Lett.* **8**, 2432-2437 (2017). https://doi.org:10.1021/acs.jpclett.7b00940

15 Yarkony, D. R. On the adiabatic to diabatic states transformation near intersections of conical intersections. *J. Chem. Phys.* **112**, 2111-2120 (2000). https://doi.org:10.1063/1.480779

16 Fdez. Galván, I., Delcey, M. l. G., Pedersen, T. B., Aquilante, F. & Lindh, R. Analytical state-average complete-active-space self-consistent field nonadiabatic coupling vectors: Implementation with density-fitted two-electron integrals and application to conical intersections. *J. Chem. Theory Comput.* **12**, 3636-3653 (2016).

17 Staroverov, V. N. & Davidson, E. R. Diradical Character of the Cope Rearrangement Transition State. *Journal of the American Chemical Society* **122**, 186-187 (2000). https://doi.org:10.1021/ja993375x

18 Bochenkova, A. V. *et al.* Mechanism of resonant electron emission from the deprotonated GFP chromophore and its biomimetics. *Chem. Sci.* **8**, 3154-3163 (2017).
